# A Novel “Off-On” Fluorescent Probe Based on Carbon Nitride Nanoribbons for the Detection of Citrate Anion and Live Cell Imaging

**DOI:** 10.3390/s18041163

**Published:** 2018-04-11

**Authors:** Yanling Hu, Dongliang Yang, Chen Yang, Ning Feng, Zhouwei Shao, Lei Zhang, Xiaodong Wang, Lixing Weng, Zhimin Luo, Lianhui Wang

**Affiliations:** 1 Key Laboratory for Organic Electronics & Information Displays (KLOEID) and Jiangsu Key Laboratory for Biosensors, Institute of Advanced Materials (IAM), Nanjing University of Posts and Telecommunications, 9 Wenyuan Road, Nanjing 210023, China; huyanling124@hotmail.com (Y.H.); yangdl1023@163.com (D.Y.); 1015061531@njupt.edu.cn (C.Y.); 18705196782@163.com (N.F.); 18305167897@163.com (Z.S.); iamlzhang@njupt.edu.cn (L.Z.); 18851825985@163.com (X.W.); iamzmluo@njupt.edu.cn (Z.L.); 2 College of Geography and Biological Information, Nanjing University of Posts and Telecommunications, 9 Wenyuan Road, Nanjing 210023, China; lxweng@njupt.edu.cn

**Keywords:** carbon nitride, nanoribbons, fluorescent detection, citrate anion, biosensing

## Abstract

A novel fluorescent “off-on” probe based on carbon nitride (C_3_N_4_) nanoribbons was developed for citrate anion (C_6_H_5_O_7_^3−^) detection. The fluorescence of C_3_N_4_ nanoribbons can be quenched by Cu^2+^ and then recovered by the addition of C_6_H_5_O_7_^3−^, because the chelation between C_6_H_5_O_7_^3−^ and Cu^2+^ blocks the electron transfer between Cu^2+^ and C_3_N_4_ nanoribbons. The turn-on fluorescent sensor using this fluorescent “off-on” probe can detect C_6_H_5_O_7_^3−^ rapidly and selectively, showing a wide detection linear range (1~400 μM) and a low detection limit (0.78 μM) in aqueous solutions. Importantly, this C_3_N_4_ nanoribbon-based “off-on” probe exhibits good biocompatibility and can be used as fluorescent visualizer for exogenous C_6_H_5_O_7_^3−^ in HeLa cells.

## 1. Introduction

Citrate is a critical metabolite that is involved in various biological systems, such as mitochondrial energy generation, cytosolic biomacromolecular synthesis, inflammatory response, blood coagulation, and the regulation of the size of apatite crystals in bone [1,2,3,4,5]. Citrate deficiency is the main reason for kidney dysfunction such as nephrocalcinosis and nephrolithiasis [6]. Recent medical research has shown that the tracking of citrate levels has become an effective method for the identification of prostate cancer [2,7]. Therefore, monitoring citrate content is of great importance in biomedical and analytical sciences. To date, many analytical methods have been used for citrate detection including electrochemistry [8], capillary electrophoresis [9], polarography [10], gas or liquid chromatography [11,12], UV-vis spectrophotometry [6,13], and spectrofluorimetry [14,15]. The spectrofluorimetry method has attracted great attention owing to its easy operation, high sensitivity, and lower equipment requirements [6,13,16].

Carbon nitride (C_3_N_4_) is a metal-free polymeric semi-conductor with a narrow bandgap of about 2.7 eV. Due to its special photoluminescence (PL), facile synthesis, and good biocompatibility [17,18], C_3_N_4_ has been applied for environmental decontamination, artificial photosynthesis, biotherapy, bioimaging, and sensors [17,18,19,20,21,22,23,24]. So far, some fluorescent sensing platforms based on C_3_N_4_ have been designed for the detection of various ions and molecules, including Cu^2+^, Fe^3+^, Ag^+^, acetylthiocholine, biothiols, and cyanide [18,23,25,26]. However, to the best of our knowledge, there is no report of fluorescent sensors based on C_3_N_4_ nanoribbons for citrate anion detection.

**Scheme 1:**
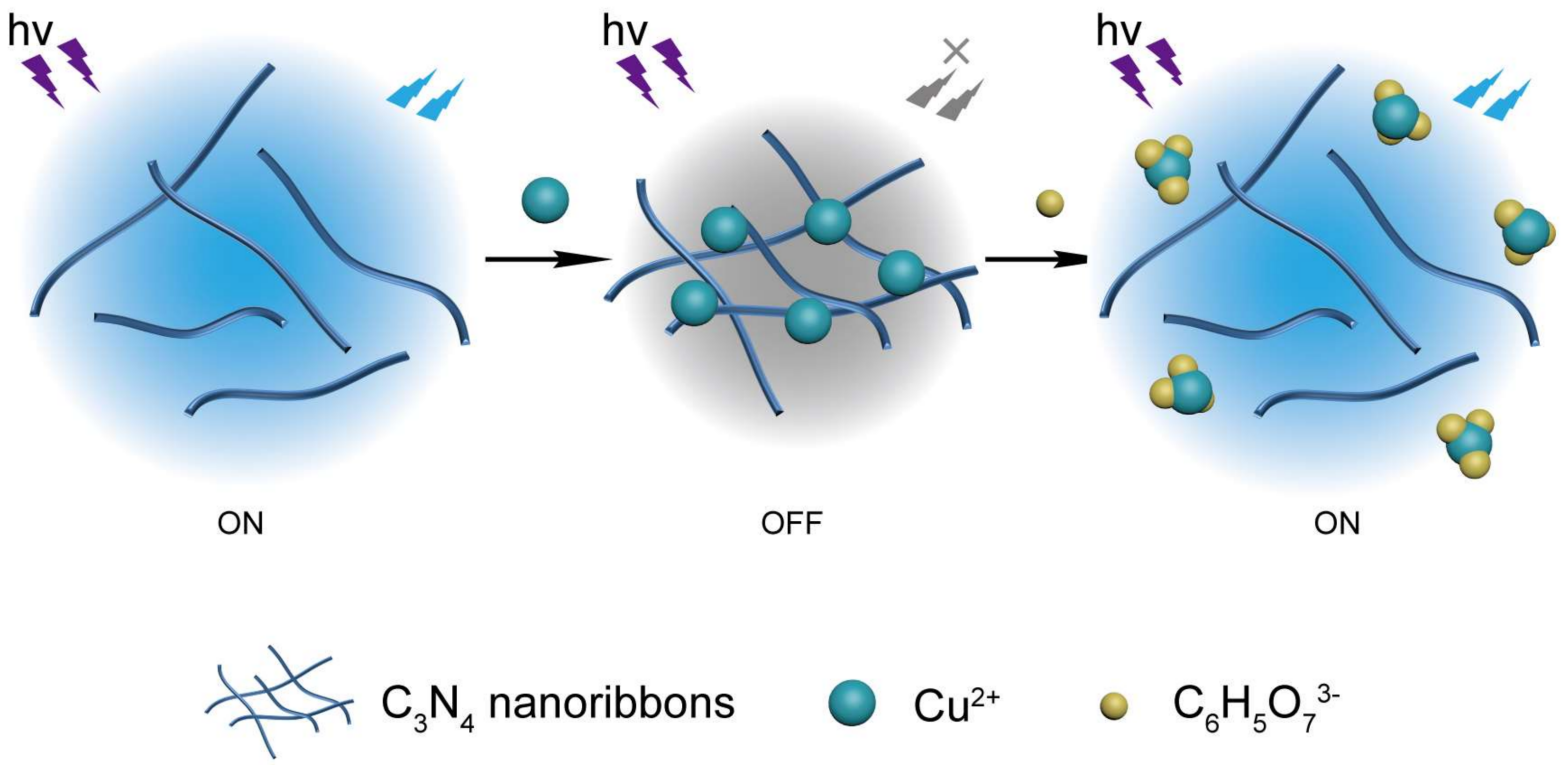
Schematic illustration of the C_3_N_4_ nanoribbon-based fluorescent citrate sensor.

In this work, blue fluorescent C_3_N_4_ nanoribbons were prepared by alkali-catalyzed hydrolysis from bulk C_3_N_4_ [27]. Furthermore, we demonstrated that the obtained C_3_N_4_ nanoribbons can serve as a novel “off-on” fluorescent probe for the detection of citrate anion (C_6_H_5_O_7_^3−^) with excellent sensitivity and selectivity based on fluorescence quenching by Cu^2+^ through a photoinduced electron transfer [28,29] and fluorescence recovery by the addition of C_6_H_5_O_7_^3−^. We hypothesized that the interaction of Cu^2+^ and C_3_N_4_ nanoribbons is inhibited by the strong chelation between Cu^2+^ and C_6_H_5_O_7_^3−^ [30,31,32]. This is the first time that C_3_N_4_ nanoribbons have been applied for C_6_H_5_O_7_^3−^ detection. Importantly, the turn-on fluorescent sensor using this fluorescent “off-on” probe exhibits low cytotoxicity and can be applied for C_6_H_5_O_7_^3−^ detection in living cells. Scheme 1 illustrates the sensing principle of the C_3_N_4_ nanoribbon-based fluorescent sensor.

## 2. Materials and Methods

### 2.1. Materials and Reagents

Dulbecco’s modified Eagle’s medium (DMEM), fetal bovine serum, and 3-(4,5-dimethyl-thiazol-2-yl)-2,5-diphenyltetrazolium bromide (MTT) were purchased from Hyclone. HeLa cells were supplied by KeyGen Biotech Co., Ltd. (Nanjing, China). All inorganic salts were of analytical grade and obtained from Aladdin reagent (Shanghai, China). Formic acid, sodium acetate, and propionic acid were purchased from Sinopharm Chemical Reagent Co., Ltd. (Beijing, China). Ultrapure water (Milli-Q system, Millipore Corp., Billerica, MA, USA) was used throughout this study.

### 2.2. Characterization

UV–vis spectroscopy measurements were performed on a Shimadzu UV-3600 spectrophotometer. Fluorescence spectra were recorded on an RF-5301PC spectrofluorophotometer. The Fourier transform infrared (FTIR) spectrum of C_3_N_4_ nanoribbons was measured on a Nexus 870 FTIR spectrometer. A JEOL 2010 transmission electron microscope (TEM) was used for the characterization of C_3_N_4_ nanoribbons. The X-ray diffraction (XRD) pattern of C_3_N_4_ nanoribbons was recorded on an X-ray powder diffractometer with graphite monochromatized Cu Kα radiation (D8 Advance; Bruker, Karlsruhe, Germany). The X-ray photoelectron spectroscopy (XPS) investigation was carried out on PHI 5000 VersaProbe system with Al cathode as the X-ray source. The C_3_N_4_ nanoribbons was dropped on silicon slices for XPS characterization. Dynamic light scattering (DLS) and zeta potential measurements were conducted on a zeta potential analyzer (Zeta PALS, Brookhaven Instruments Corp., Brookhaven, NY, USA). Cell fluorescent images were recorded by confocal laser scanning microscopy (Olympus FV1000, Tokyo, Japan).

### 2.3. Preparation of C_3_N_4_ Nanoribbons

The bulk C_3_N_4_ was prepared by the calcination of melamine at 600 °C (5.0 °C min^−1^) for 4 h under an Ar atmosphere [33]. The C_3_N_4_ nanoribbons were prepared via the alkali-catalyzed hydrolysis of bulk C_3_N_4_ [27]. In brief, 10 mg of bulk C_3_N_4_ was dispersed in 10 mL of sodium hydroxide solution (8.0 M) and sonicated for 2 h at 60 °C. After cooling to room temperature, this solution was centrifuged and washed five times with ultrapure water. The product was collected and dialyzed (the molecular weight cutoff of the dialysis bag was 1000 kDa) for further experiments.

### 2.4. Synthesis of Cu^2+^-C_3_N_4_ Nanoribbon Complex

One hundred microliters of CuCl_2_ (10 mM) was added to 9.9 mL of C_3_N_4_ nanoribbons aqueous solution (1 mg mL^−1^). Then, the mixture was stirred for 10 min at room temperature. After that, the mixture was centrifuged and washed with ultrapure water to remove excessive copper ions. Finally, the obtained precipitate was dispersed in ultrapure water to obtain a Cu^2+^-C_3_N_4_ nanoribbon solution.

### 2.5. Fluorescent Detection of C_6_H_5_O_7_^3−^

Forty microliters of Cu^2+^-C_3_N_4_ nanoribbon complex (C_3_N_4_: 1 mg mL^−1^) was added to 960 μL ultrapure water, and then C_6_H_5_O_7_^3−^ solution with various concentrations was added to the Cu^2+^-C_3_N_4_ nanoribbon complex. After standing for 20 s, the fluorescence spectra were monitored at the excitation of 360 nm.

### 2.6. Selectivity of Cu^2+^-C_3_N_4_ Nanoribbon-Based Probe for C_6_H_5_O_7_^3−^ Detection

To explore the possible interference of other anions, formic acid, sodium acetate, propionic acid and the following anionic sodium/potassium salts were used in this study: 1 mM anion solutions, including Br^−^, C_6_H_5_O_7_^3−^, Cl^−^, CN^−^, F^−^, H_2_PO_4_^−^, HCO_3_^−^, I^−^, NO_3_^−^, OH^−^, CH_3_COO^−^, and SO_4_^2−^ were added to the solution of the Cu^2+^-C_3_N_4_ nanoribbon complex, respectively. After thoroughly mixing and standing for 20 s, the fluorescence measurements were carried out to investigate the selectivity of the proposed fluorescent sensor.

### 2.7. Cell Imaging and Cytotoxicity Assay

Human cervical cancer (HeLa) cells used in this study were cultured at 37 °C in a 5% CO_2_ incubator in DMEM medium, which contains fetal bovine serum (10%), streptomycin (100 mg mL^−1^), and penicillin (100 U mL^−1^). When the cells had grown to 80% confluency, the cells were digested with trypsin, collected and seeded in a confocal dish, and cultured overnight. Then the cells were pretreated with C_6_H_5_O_7_Na_3_ (1 mM). After 12 h incubation, the cells were gently washed with phosphate-buffered saline (PBS) solution (10 mM, pH = 7.4) and treated with Cu^2+^-C_3_N_4_ nanoribbon complex for another 4 h. Finally, the resulting cells were washed with PBS solution (10 mM, pH = 7.4) and then fluorescence images were taken on a confocal microscope under UV excitation.

For cytotoxicity assay, 100 μL of cells suspension (10^5^ cells mL^−1^) was seeded to each well of 96-well plates. Then the medium was replaced by different concentrations of C_3_N_4_ nanoribbons or Cu^2+^-C_3_N_4_ nanoribbon complex and culture for 24 h. Following this, the cells were washed with PBS solution. After that, the standard MTT assay was carried out for the determination of cell viabilities relative to the untreated cells.

## 3. Results and Discussion

### 3.1. Characterization of C_3_N_4_ Nanoribbons

**Figure 1:**
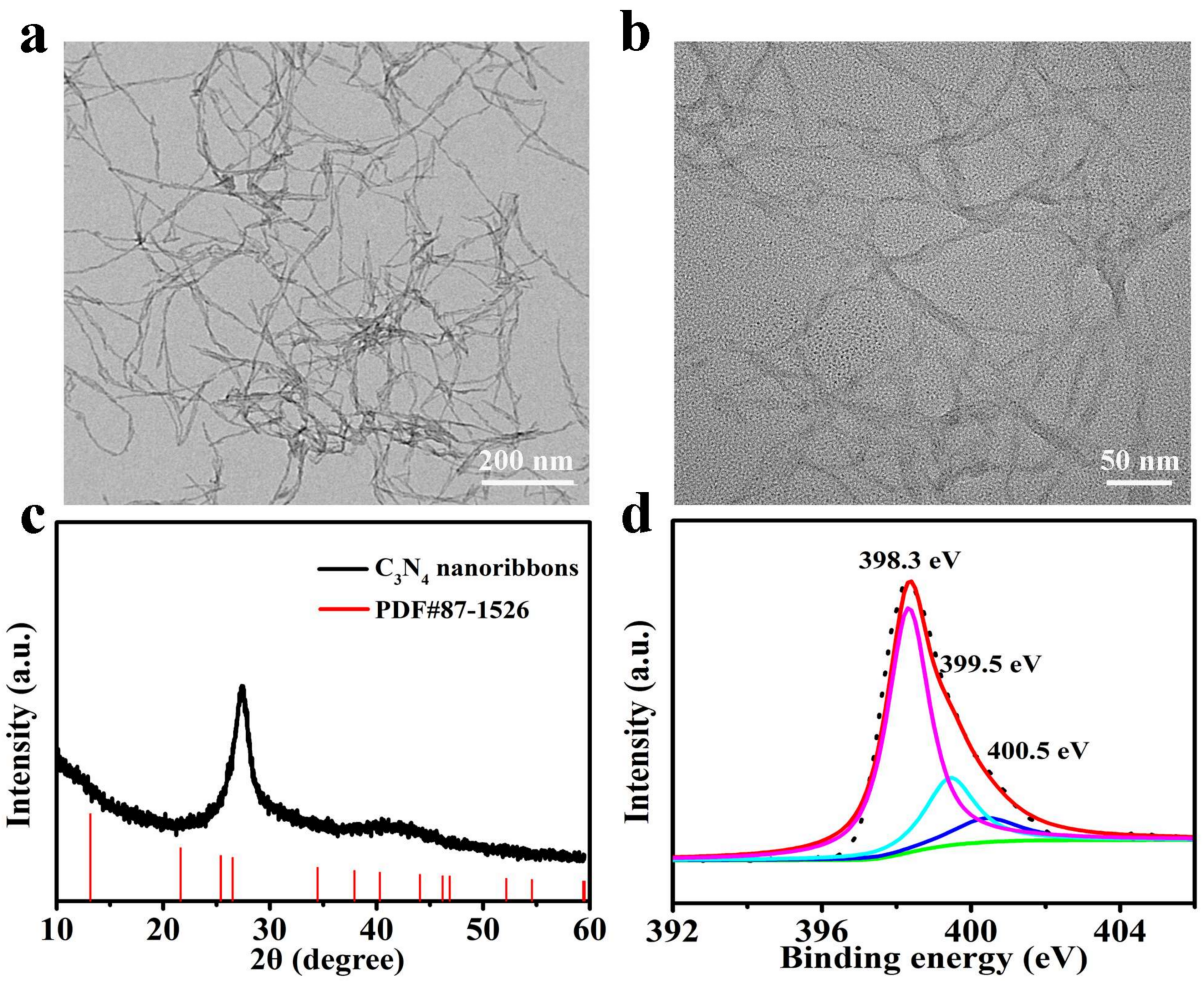
(**a**) TEM image, (**b**) HRTEM image, (**c**) X-ray diffraction (XRD) pattern and (**d**) N 1s X-ray photoelectron spectroscopy (XPS) spectrum of C_3_N_4_ nanoribbons.

The C_3_N_4_ nanoribbons were prepared by ultrasonic exfoliation of bulk C_3_N_4_ in an alkaline solution. The related characterizations of bulk C_3_N_4_ are shown in Figure S1. TEM images display the morphology and size distribution of the C_3_N_4_ nanoribbons. As shown Figure 1a, C_3_N_4_ nanoribbons present an average diameter of approximately 5 nm and a length of up to 200 nm. High-resolution transmission electron microscopy (HRTEM) image clearly shows that single and few-layer C_3_N_4_ nanoribbons were obtained (Figure 1b). The XRD pattern of C_3_N_4_ nanoribbons (Figure 1c) showed a broad distinct diffraction peak at 27.2°, which can be ascribed to the strong π-conjugated layers characteristic (002) of C_3_N_4_ [17,34].

The composition and structure of C_3_N_4_ nanoribbons were confirmed by XPS and FTIR measurements. The XPS survey spectrum of C_3_N_4_ nanoribbons (Figure S2) displays binding energies of C (283 eV) and N (397 eV). The C 1s XPS spectrum of C_3_N_4_ nanoribbons (Figure S3) can be fitted into three peaks centering at 284.6, 285.4, and 287.8 eV, which can be attributed to C-C, sp^2^C=N, and sp^3^C-N of C_3_N_4_, respectively [35,36,37,38,39]. The XPS spectrum of the N 1s spectrum (Figure 1d) can be fitted into three different peaks at 398.3, 399.5, and 400.5 eV, being assigned to C=N-C, (N-(C)_3_), and -NH_2_, respectively [36,40]. The FTIR spectrum of C_3_N_4_ nanoribbons is presented in Figure S4. The broad peaks at 3330 and 3182 cm^−1^ are ascribed to the stretching vibrations of NH_2_ and N-H groups, respectively [39]. The peaks centered at 1637, 1577, 1420, 1334, and 1284 cm^−1^ indicated the typical stretching modes of CN heterocycles [41,42,43]. Beside the peaks mentioned above, the peak at 810 cm^−1^ indicated the vibration of the *s*-triazine ring [34,44].

**Figure 2:**
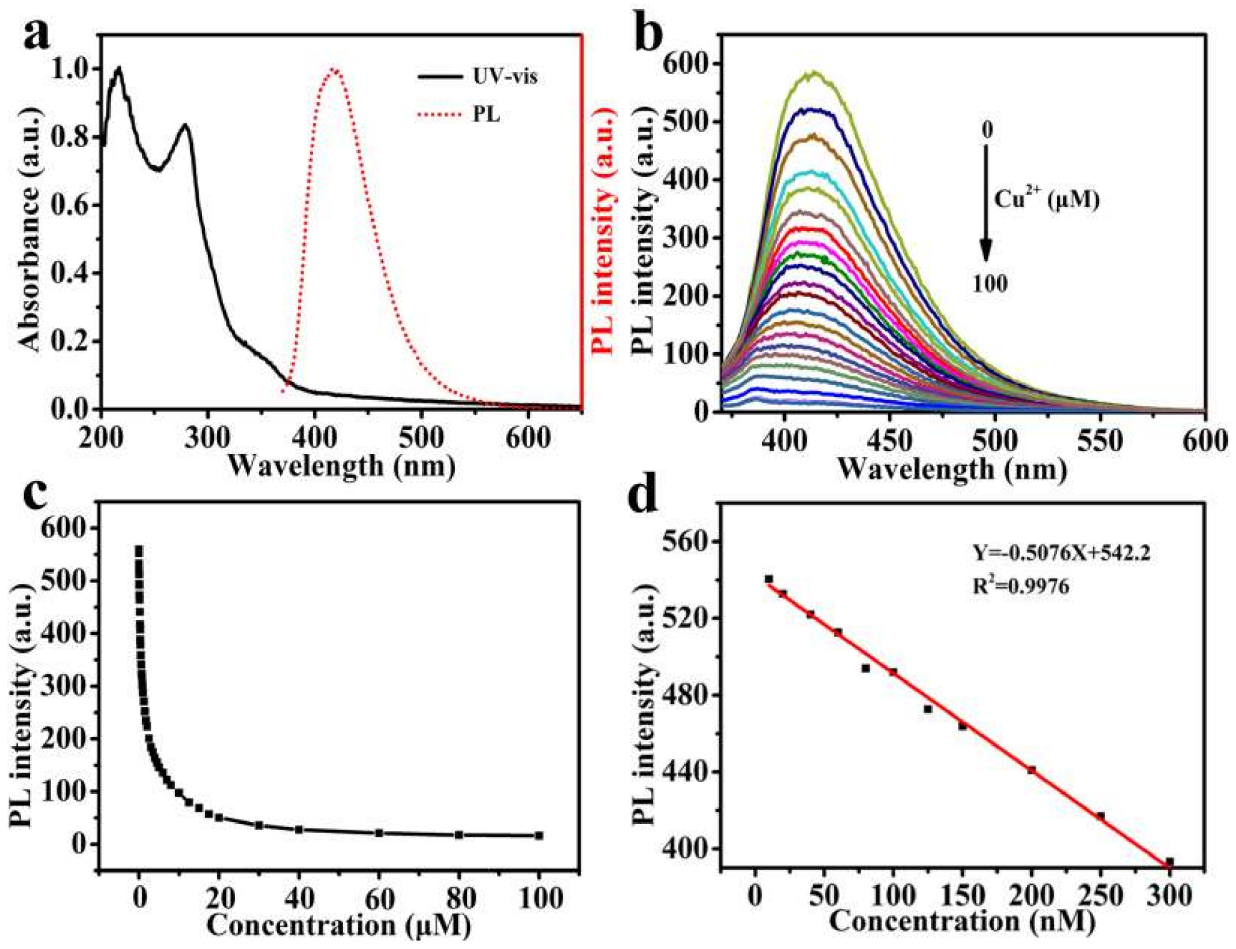
(**a**) UV-vis and photoluminescence (PL) spectra of C_3_N_4_ nanoribbons. (**b**) The PL spectra of C_3_N_4_ nanoribbons (40 μg mL^−1^) in the presence of different concentrations of Cu^2+^. (**c**) PL intensity responses of C_3_N_4_ nanoribbons with varying concentrations of Cu^2+^ in an aqueous solution. (**d**) The linear calibration of PL intensity versus the concentrations of Cu^2+^.

The photophysical properties of C_3_N_4_ nanoribbons were investigated by UV-vis and PL spectra (Figure 2a). The prepared C_3_N_4_ nanoribbons present two strong absorption peaks at 216 and 278 nm. Meanwhile, a fluorescent emission at 415 nm can be seen from fluorescence spectrum at the excitation of 360 nm (Figure 2a). The PL intensity of C_3_N_4_ nanoribbons increases dramatically with the increasing pH of solution ranging from 9 to 12, and displays slight changes under acid conditions (Figure S5).

### 3.2. The Influence of Metal Ions on the Fluorescence of C_3_N_4_ Nanoribbons

A previous study indicated that Cu^2+^, Fe^3+^, and Ag^+^ can quench the fluorescence of C_3_N_4_ due to the photoinduced electron transfer from C_3_N_4_ to metal ions [23,25,45]. In this experiment, the fluorescent responses of C_3_N_4_ nanoribbons towards different metal ions were investigated. As shown in Figure S6, C_3_N_4_ nanoribbons display a slight change of the PL intensity in the presence of Al^3+^, Ba^2+^, K^+^, Li^+^, Mg^2+^, Na^+^, Pb^2+^, Sn^2+^, and Zn^2+^ (100 μM). However, the PL intensity of C_3_N_4_ nanoribbons dramatically decreases with the addition of Cu^2+^, Ag^+^, Fe^3+^, Co^2+^, Mn^2+^, and Ni^2+^, especially for Cu^2+^ and Ag^+^. Cu^2+^ was chosen as the quencher in this work [36]. A decrease of PL intensity of C_3_N_4_ nanoribbons is observed as the concentration of Cu^2+^ increases (Figure 2b,c), and it is almost completely quenched after the addition of 40 μM Cu^2+^. Figure 2d shows that the PL intensity of C_3_N_4_ at 415 nm versus the concentrations of Cu^2+^ exhibits a linear relationship ranging from 10 to 300 nM (R^2^ = 0.9976).

### 3.3. Sensitivity and Selectivity of the Fluorescent “Off-On” Probe Based on C_3_N_4_ Nanoribbons for C_6_H_5_O_7_^3−^ Detection

The influence of incubation time on the fluorescence recovery of C_3_N_4_ nanoribbons was measured. As Figure S7 displayed, the PL intensity of Cu^2+^-C_3_N_4_ nanoribbon complex increases rapidly with the time after the addition of C_6_H_5_O_7_^3−^, and maintains a plateau after 20 s. Therefore, an incubation time of 20 s was selected for subsequent experiments.

**Figure 3:**
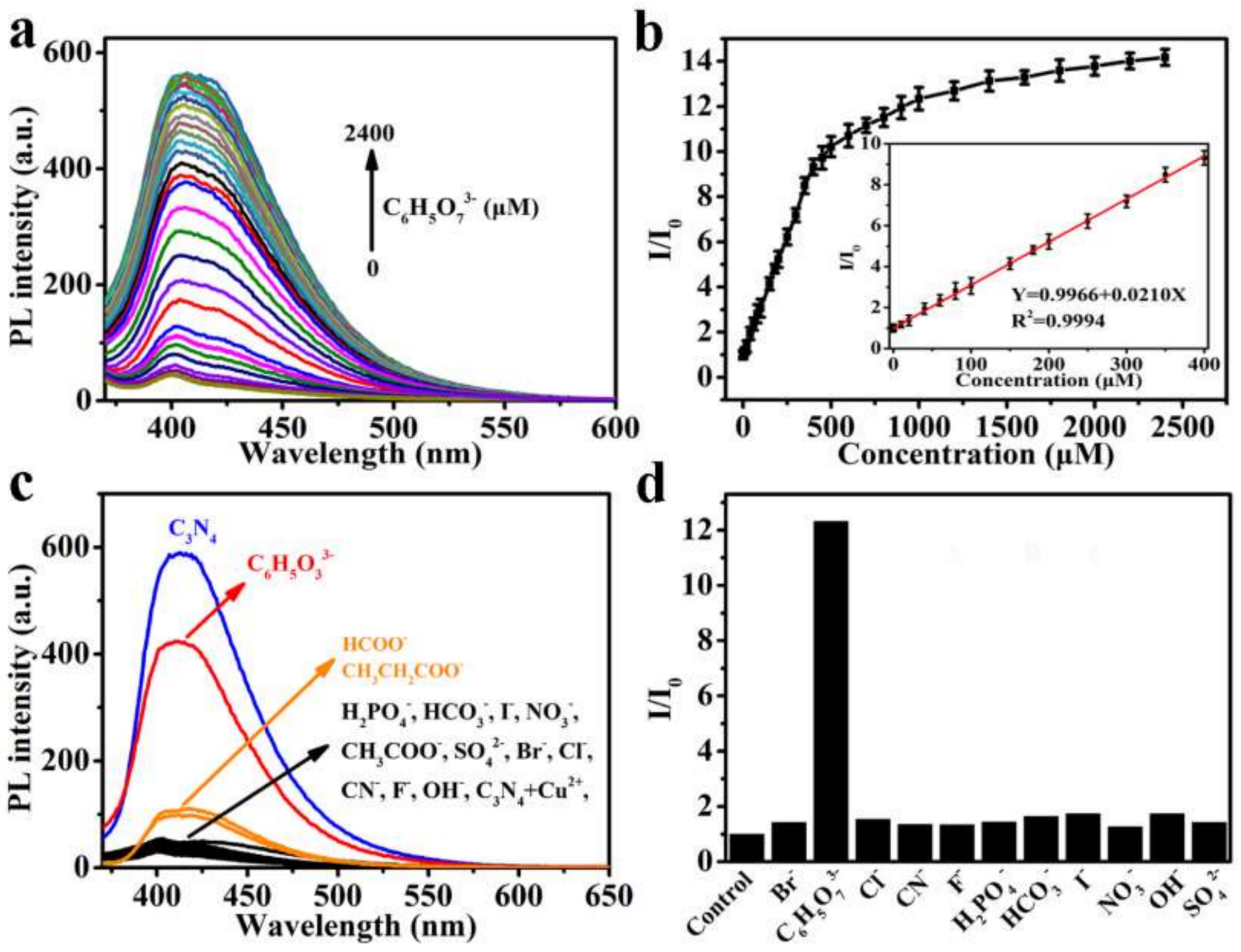
(**a**) Fluorescence spectra of Cu^2+^-C_3_N_4_ nanoribbon complex with increasing concentrations of C_6_H_5_O_7_^3−^. (**b**) Plot of the fluorescence enhancement (I/I_0_) of the Cu^2+^-C_3_N_4_ nanoribbon complex after the addition of different concentrations of C_6_H_5_O_7_^3−^. The linear calibration range from 1 to 400 μM is shown as an inset. (**c**) Fluorescence spectra of the Cu^2+^-C_3_N_4_ nanoribbon complex in the presence of Br^−^, C_6_H_5_O_7_^3−^, Cl^−^, CN^−^, F^−^, H_2_PO_4_^−^, HCO_3_^−^, I^−^, NO_3_^−^, OH^−^, SO_4_^2−^, HCOO^−^, CH_3_COO^−^, and CH_3_CH_2_COO^−^ (1 mM). (**d**) The value of fluorescent enhancement (I/I_0_) of the Cu^2+^-C_3_N_4_ nanoribbon complex after the addition of Br^−^, C_6_H_5_O_7_^3−^, Cl^−^, CN^−^, F^−^, H_2_PO_4_^−^, HCO_3_^−^, I^−^, NO_3_^−^, OH^−^, and SO_4_^2−^ (1 mM). I_0_ and I stand for the fluorescence intensities of Cu^2+^-C_3_N_4_ nanoribbon complex at 415 nm in the absence and presence of different anions, respectively.

As is well known, sensitivity is a critical parameter to assess the sensing performance [46,47]. The sensitivity of the fluorescent sensor using the C_3_N_4_ nanoribbon-based “off-on” probe was evaluated. The PL intensity of the Cu^2+^-C_3_N_4_ nanoribbon complex with different concentrations of C_6_H_5_O_7_^3−^ was recorded. As illustrated in Figure 3a,b, the PL intensity of the Cu^2+^-C_3_N_4_ nanoribbon complex was obviously recovered as the concentration of C_6_H_5_O_7_^3−^ increased. The PL intensity of the Cu^2+^-C_3_N_4_ nanoribbon complex was completely recovered with the addition of C_6_H_5_O_7_^3−^ (2.25 mM). The fluorescent sensor using the C_3_N_4_ nanoribbon-based “off-on” probe shows a linear range from 1 to 400 μM and the calculated detection limit is 0.78 μM (S/N = 3) (inset in Figure 3b). Compared with previously reported fluorescent probes, such as coumarin [6], rhodamine [13], diketoprrrolopyrrole [15], TPIOP-boronate [48], and CdTe quantum dots [14], the fluorescent sensor using the C_3_N_4_ nanoribbon-based “off-on” probe exhibits better sensing performance for C_6_H_5_O_7_^3−^ detection with a broader linear range and faster response time (Table S1) [6,14,15,49].

To investigate the selectivity of the Cu^2+^-C_3_N_4_ nanoribbon-based probe towards C_6_H_5_O_7_^3−^, the fluorescence responses of the Cu^2+^-C_3_N_4_ nanoribbon complex towards other anions (Br^−^, Cl^−^, CN^−^, F^−^, H_2_PO_4_^−^, HCO_3_^−^, I^−^, NO_3_^−^, OH^−^, and SO_4_^2−^) was measured. As shown in Figure 3c,d, most of the tested anions almost do not affect the PL intensity of the Cu^2+^-C_3_N_4_ nanoribbon complex_,_ while C_6_H_5_O_7_^3−^ can recover the fluorescence of C_3_N_4_ nanoribbons effectively. Moreover, formic acid, sodium acetate, and propionic acid were also applied for the selectivity analysis of the proposed detection method (Figure S8), showing that the fluorescence presented the highest recovery in the presence of C_6_H_5_O_7_^3−^. Furthermore, the PL responses of the Cu^2+^-C_3_N_4_ nanoribbon-based probe towards C_6_H_5_O_7_^3−^ were also investigated under different pH levels and metal ions environments. As shown in Figure S9a, the fluorescence intensity of the Cu^2+^-C_3_N_4_ nanoribbon-based probe with or without C_6_H_5_O_7_^3−^ did not show obvious change in the pH range from 4 to 8. More importantly, almost all of the cations (10 μM) did not affect the fluorescence response of the Cu^2+^-C_3_N_4_ nanoribbon-based probe, except for Ag^+^ (Figure S9b). Further, cell lysate (1 × 10^6^ cell mL^−1^) and biological molecules (10 μM), including glutamic acid, ascorbic acid, glutathione, and DNA, were introduced to examine the probe’s stability. As Figure S9c shows, except for glutathione, the Cu^2+^-C_3_N_4_ nanoribbon-base probe exhibited no obvious fluorescence change upon the addition of C_6_H_5_O_7_^3−^ and a biological molecule (10 μM) mixture, even in cell lysate. These results indicate that the Cu^2+^-C_3_N_4_ nanoribbon-based probe can be used for C_6_H_5_O_7_^3−^ detection in more complex environments.

The mechanism of the fluorescence quenching and recovering process were studied by DLS and zeta-potential for this system (Figure S10). The average hydrodynamic size of C_3_N_4_ nanoribbons increased from 230 to 1523 nm in the presence of Cu^2+^ along with fluorescence quenching. A change of the zeta-potential from −20.6 mV to −10.4 mV was observed after the C_3_N_4_ nanoribbons interacted with Cu^2+^. This result indicates that a non-fluorescent complex (Cu^2+^-C_3_N_4_ nanoribbon) was formed [50]. With the addition of C_6_H_5_O_7_^3−^, the hydrodynamic size decreased because of the chelation between Cu^2+^ and C_6_H_5_O_7_^3−^, indicating the release of Cu^2+^ from C_3_N_4_ nanoribbons and resulting in the restoration of the fluorescence of C_3_N_4_ nanoribbons [18,19,40]. In order to rule out the effect of nonspecific binding, the PL intensity of the C_3_N_4_ nanoribbons was monitored after the addition of different anion solutions. The result shows that, except for OH^−^, the other anions did not dramatically affect the PL intensity of the C_3_N_4_ nanoribbons (Figure S11).

### 3.4. Intracellular Imaging of C_6_H_5_O_7_^3−^

**Figure 4:**
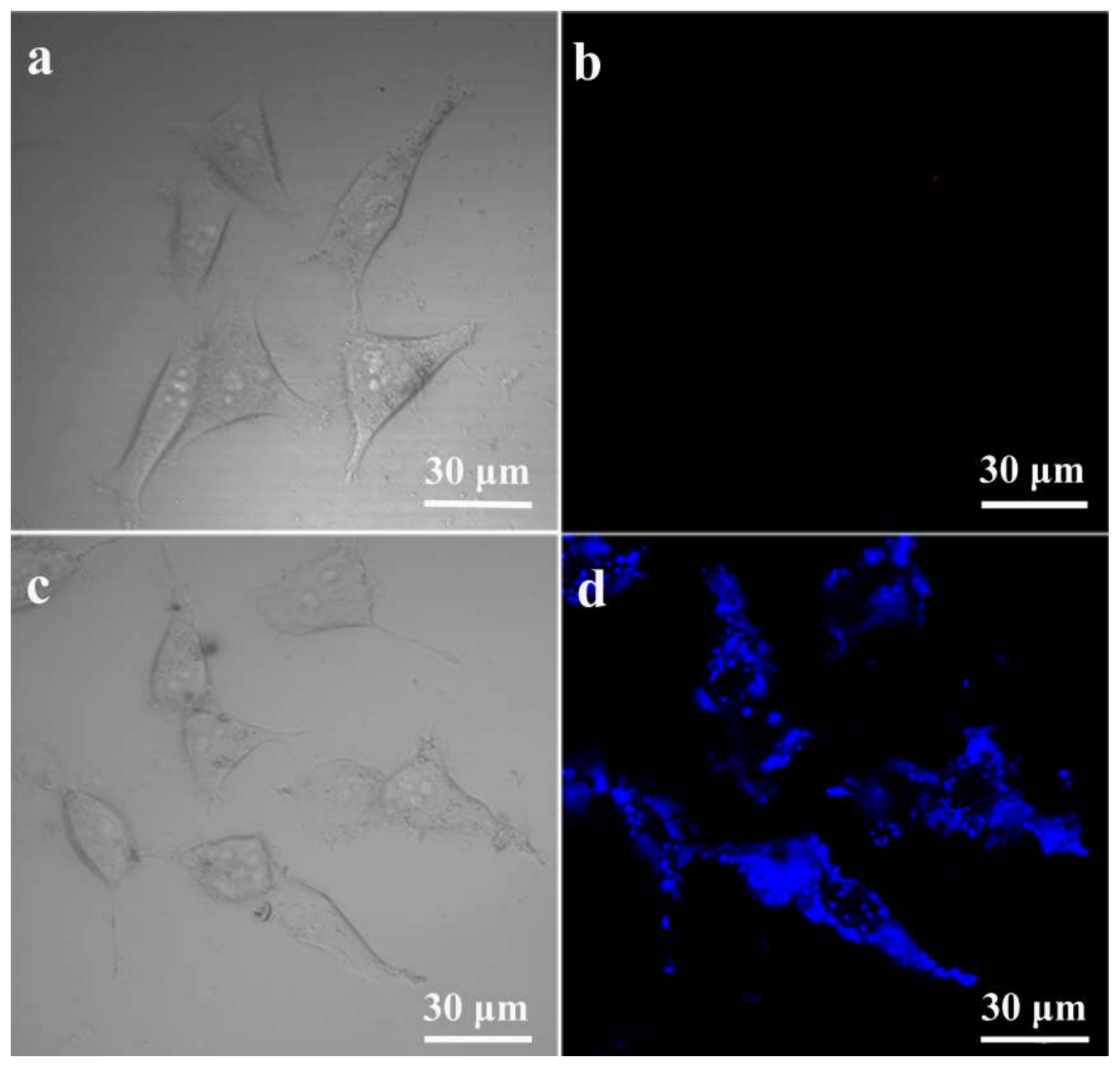
(**a**) Bright field and (**b**) fluorescent images of HeLa cells incubated with the Cu^2+^-C_3_N_4_ nanoribbon complex for 4 h. (**c**) Bright field and (**d**) fluorescent images of HeLa cells pretreated with C_6_H_5_O_7_Na_3_ (1 mM) for 12 h and then incubated with the Cu^2+^-C_3_N_4_ nanoribbon complex for 4 h.

For further biological applications, the cytotoxicity of the C_3_N_4_ nanoribbons and Cu^2+^-C_3_N_4_ nanoribbon complex to HeLa cells was assessed through MTT assays. As shown in Figure S12, the viability of HeLa cells showed no obvious change after incubation with the C_3_N_4_ nanoribbons or Cu^2+^-C_3_N_4_ nanoribbon complex for 24 h, indicating their good biocompatibility. Since the Cu^2+^-C_3_N_4_ nanoribbon complex showed a highly selective and sensitive response towards C_6_H_5_O_7_^3−^, this novel fluorescent “off-on” probe based on C_3_N_4_ nanoribbons might be potentially applied for the intracellular detection of C_6_H_5_O_7_^3−^. As shown in Figure 4, bright blue fluorescence was observed in C_6_H_5_O_7_^3−^-overloaded HeLa cells incubated with the Cu^2+^-C_3_N_4_ nanoribbon complex, whereas no fluorescence was detected in the control HeLa cells incubated with the Cu^2+^-C_3_N_4_ nanoribbon complex. Besides, Z-scan fluorescent images of living HeLa cells further confirmed that the Cu^2+^-C_3_N_4_ nanoribbon-based probe can be taken up by the cells (Figure S9d). These results demonstrate that the Cu^2+^-C_3_N_4_ nanoribbon complex is membrane permeable and could be used as a fluorescent visualizer for the intracellular imaging of C_6_H_5_O_7_^3−^.

## 4. Conclusions

In summary, a novel fluorescent “off-on” probe based on C_3_N_4_ nanoribbons was developed for C_6_H_5_O_7_^3−^ detection. The fluorescence of C_3_N_4_ nanoribbons can be quenched by Cu^2+^ and then recovered by the addition of C_6_H_5_O_7_^3−^. The sensor using this fluorescent “off-on” probe showed a good detection linear range (1~400 μM) with a low detection limit (0.78 μM) as well as high selectivity in aqueous solutions. More importantly, this “off-on” probe based on C_3_N_4_ nanoribbons exhibited good biocompatibility and low cytotoxicity in cell environments and can be utilized for intracellular imaging of C_6_H_5_O_7_^3−^.

## Supplementary Materials

The following are available online at http://www.mdpi.com/1424-8220/18/4/1163/s1. Figure S1: (a) SEM image, (b) XPS survey spectrum, (c) XRD pattern and (d) FTIR spectrum of the bulk C_3_N_4_. Figure S2: XPS survey spectrum of C_3_N_4_ nanoribbons. Figure S3: C 1s XPS spectrum of C_3_N_4_ nanoribbons. Figure S4: FTIR spectrum of C_3_N_4_ nanoribbons. Figure S5: Effect of the pH value on the PL intensity of C_3_N_4_ nanoribbons. Figure S6: The fluorescence responses of C_3_N_4_ nanoribbons to various metal ions (Cu^2+^, Al^3+^, Ba^2+^, Co^2+^, Ag^+^, Fe^3+^, K^+^, Li^+^, Mg^2+^, Mn^2+^, Na^+^, Ni^2+^, Pb^2+^, Sn^2+^, and Zn^2+^) at a concentration of 100 μM in an aqueous solution. Figure S7: The fluorescent changes of Cu^2+^-C_3_N_4_ nanoribbon complex as a function of interaction time after the addition of C_6_H_5_O_7_^3−^ (1 mM). Figure S8: The value of the fluorescent enhancement (I/I_0_) of Cu^2+^-C_3_N_4_ nanoribbon complex after the addition of C_6_H_5_O_7_^3−^, formic acid, sodium acetate, and propionic acid. I_0_ and I are the fluorescence intensities of the Cu^2+^-C_3_N_4_ nanoribbon complex at 415 nm in the absence and presence of different anions, respectively. Figure S9: (a) Effect of the pH value on the fluorescence responses of the Cu^2+^-C_3_N_4_ nanoribbon complex after the addition of C_6_H_5_O_7_^3−^ (1 mM). (b) Fluorescence responses of the Cu^2+^-C_3_N_4_ nanoribbon complex upon the addition of C_6_H_5_O_7_^3−^ and metal ions (10 μM) mixture. (c) Fluorescence responses of the Cu^2+^-C_3_N_4_ nanoribbon complex upon the addition of C_6_H_5_O_7_^3−^ and biological molecule (10 μM) mixture. (d) Z-scan images of living HeLa cells that were preincubated with 1 mM C_6_H_5_O_7_^3−^ for 12 h and then stained with the Cu^2+^-C_3_N_4_ nanoribbon complex for 4 h. Figure S10: (a) Hydrodynamic size of C_3_N_4_ nanoribbons. (b) Hydrodynamic size of the Cu^2+^-C_3_N_4_ nanoribbon complex. (c) Hydrodynamic size of C_3_N_4_ nanoribbons after the C_6_H_5_O_7_^3−^ was added into the Cu^2+^-C_3_N_4_ nanoribbon solution. Figure S11: The fluorescence responses of C_3_N_4_ nanoribbons in an aqueous solution upon the addition of different anions (Br^−^, C_6_H_5_O_7_^3−^, Cl^−^, CN^−^, F^−^, H_2_PO_4_^−^, HCO_3_^−^, I^−^, NO_3_^−^, OH^−^, CH_3_COO^−^, and SO_4_^2−^) (final concentration: 1 mM). Figure S12: Cell viability of HeLa cells incubated with various concentrations of C_3_N_4_ nanoribbons (grey) or Cu^2+^-C_3_N_4_ nanoribbon complex (black) for 24 h. Table S1. Comparison of fluorescent citrate sensors.
