# Supplementary material for: A Novel “Off-On” Fluorescent Probe Based on Carbon Nitride Nanoribbons for the Detection of Citrate Anion and Live Cell Imaging
Source: Sensors (Basel). 2018 Apr 11;18(4):1163. doi: 10.3390/s18041163 (PMC5948658; doi:10.3390/s18041163)
Supplement: Supplementary file 1 [file sensors-18-01163-s001.pdf]

sensors

MDPI

A Novel "Off-On" Fluorescent Probe based on Carbon Nitride Nanoribbons for Detection of Citrate Anion and Live Cell Imaging

Yanling Hu, Dongliang Yang, Chen Yang, Ning Feng, Zhouwei Shao, Lei Zhang, Xiaodong Wang, Lixing Weng, Zhimin Luo, Lianhui Wang

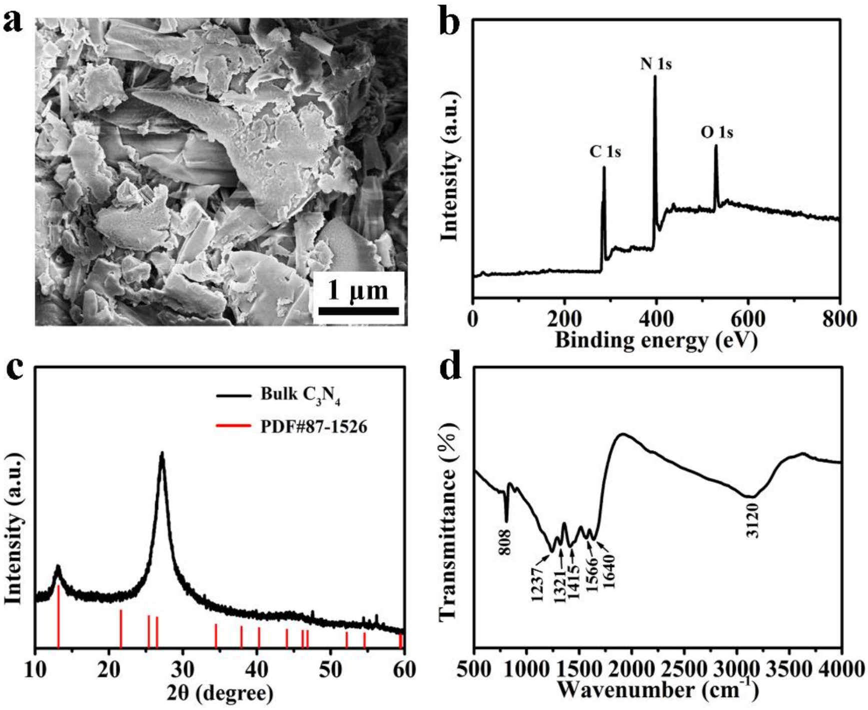

Figure S1. (**a**) SEM image, (**b**) XPS survey spectrum, (**c**) XRD pattern and (**d**) FTIR spectrum of the bulk C_3N_4.

Figure S1a shows two-dimensional bulk shape of C_3N_4. The XPS survey spectrum indicates that the bulk product contains carbon (283 eV) and nitrogen (397 eV) (Figure S1b). XRD pattern of bulk C_3N_4 (Figure S1c) shows two distinct diffraction peaks at 13.2 and 27.2°, identified as (100) due to the in-plane structural packing feature and (002) due to interlayer stacking of pi-conjugated layers [1,2]. The FTIR spectrum (Figure S1d) presents broad peaks between 3000 and 3400 cm^{-1} which are associated with the stretching vibrations of N-H groups [3]. Several strong bands of bulk C_3N_4 at 1237, 1321, 1415, 1566 and 1640 cm^{-1} belong to the typical stretching modes of CN heterocycles [4]. The peak at 808 cm^{-1} corresponds to the breathing mode of *s*-triazine [3,5].

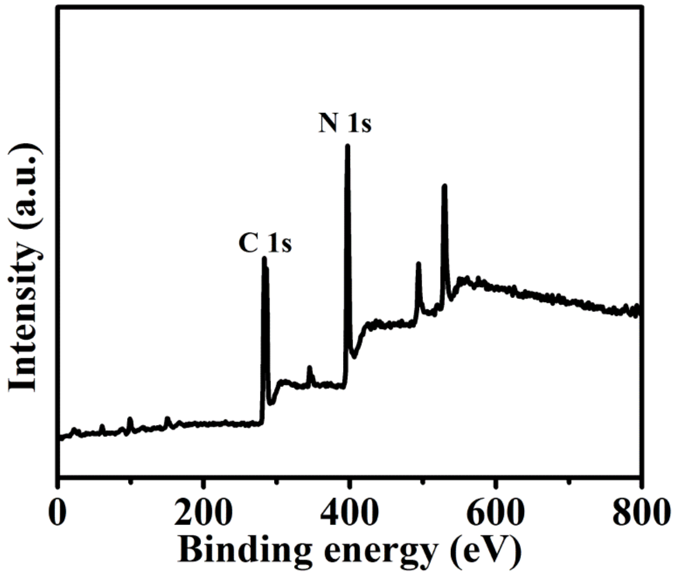

Figure S2. XPS survey spectrum of C_3N_4 nanoribbons.

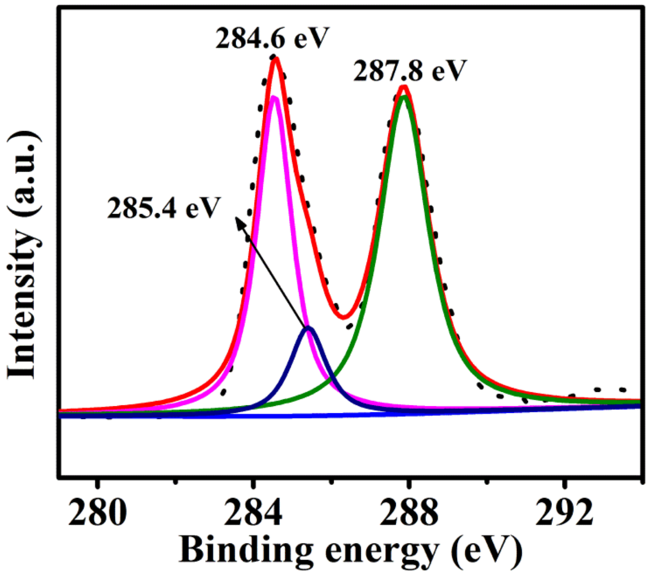

Figure S3. C 1s XPS spectrum of C_3N_4 nanoribbons.

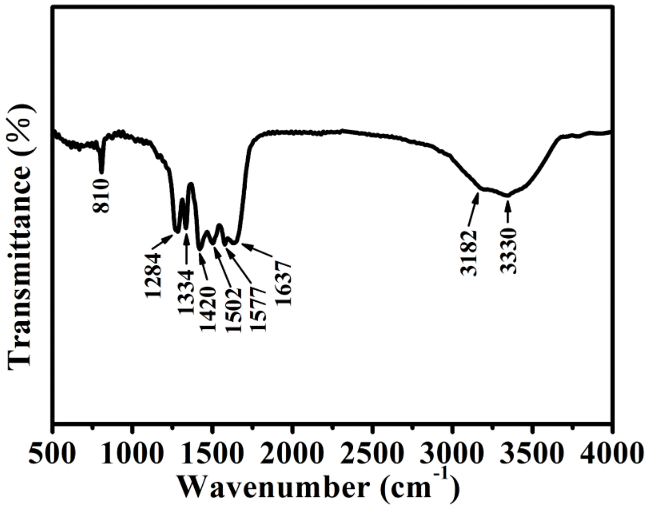

Figure S4. FTIR spectrum of C_3N_4 nanoribbons.

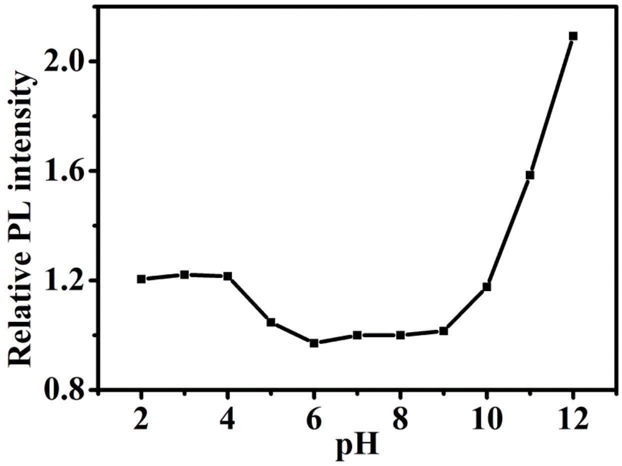

Figure S5. Effect of the pH value on the PL intensity of C_3N_4 nanoribbons.

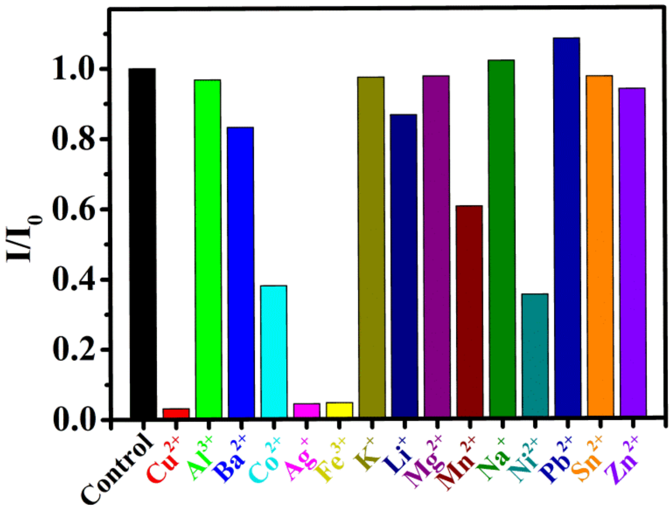

Figure S6. The fluorescence responses of C_3N_4 nanoribbons to various metal ions (Cu^{2+}, Al^{3+}, Ba^{2+}, Co^{2+}, Ag^{+}, Fe^{3+}, K^{+}, Li^{+}, Mg^{2+}, Mn^{2+}, Na^{+}, Ni^{2+}, Pb^{2+}, Sn^{2+} and Zn^{2+}) at a concentration of 100 μM in aqueous solution.

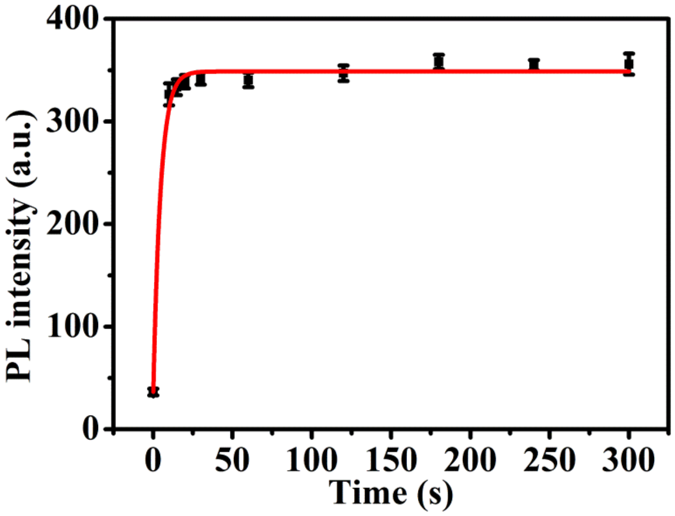

Figure S7. The fluorescent changes of Cu^{2+}-C_3N_4 nanoribbon complex as a function of interaction time after addition of $C_6H_5O_7^{3-}$ (1 mM). The fluorescence intensities were monitored at 415 nm.

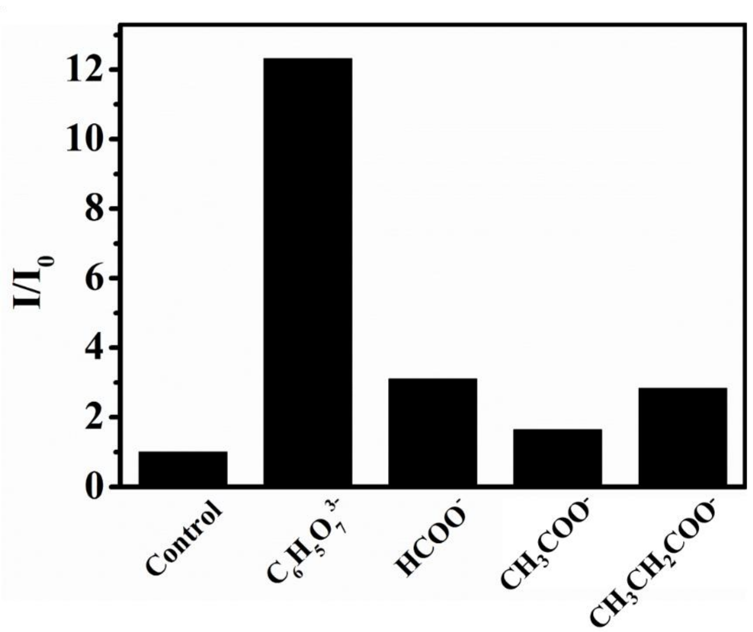

Figure S8. The value of fluorescent enhancement (I/I_0) of Cu^{2+}-C_3N_4 nanoribbon complex after the addition of $C_6H_5O_7^{3-}$, formic acid, sodium acetate and propionic acid (1 mM). I_0 and I are the fluorescence intensities of Cu^{2+}-C_3N_4 nanoribbon complex at 415 nm in the absence and presence of different anions, respectively.

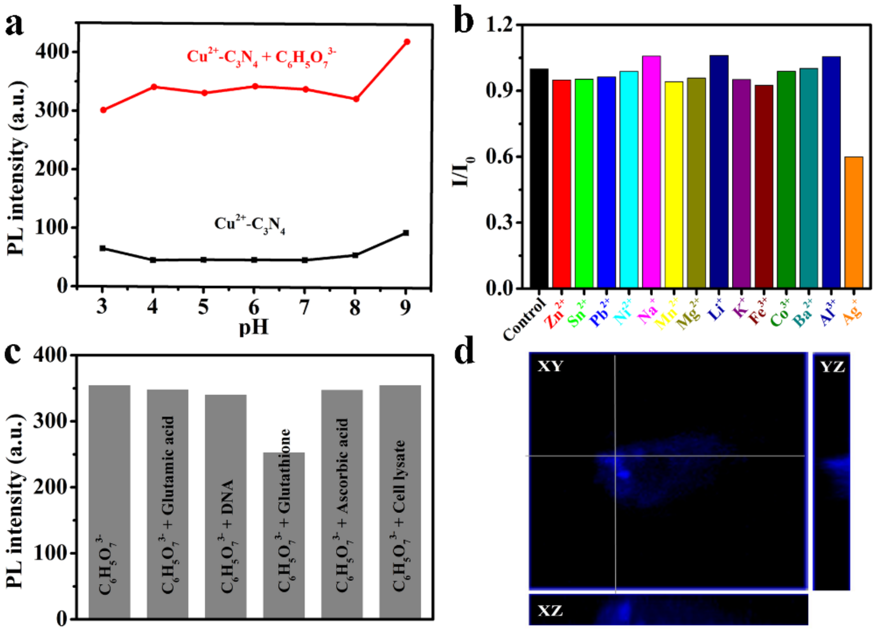

Figure S9. (**a**) Effect of the pH value on the fluorescence responses of Cu^{2+}-C_3N_4 nanoribbon complex after addition of $C_6H_5O_7^{3-}$ (1 mM). (**b**) Fluorescence responses of Cu^{2+}-C_3N_4 nanoribbon complex upon addition of $C_6H_5O_7^{3-}$ and metal ions (10 μM) mixture. (**c**) Fluorescence responses of Cu^{2+}-C_3N_4 nanoribbon complex upon addition of $C_6H_5O_7^{3-}$ and biological molecule (10 μM) mixture. (**d**) Z-scan images of living HeLa cell that preincubated with 1 mM $C_6H_5O_7^{3-}$ for 12 h and then stained with Cu^{2+}-C_3N_4 nanoribbon complex for 4 h.

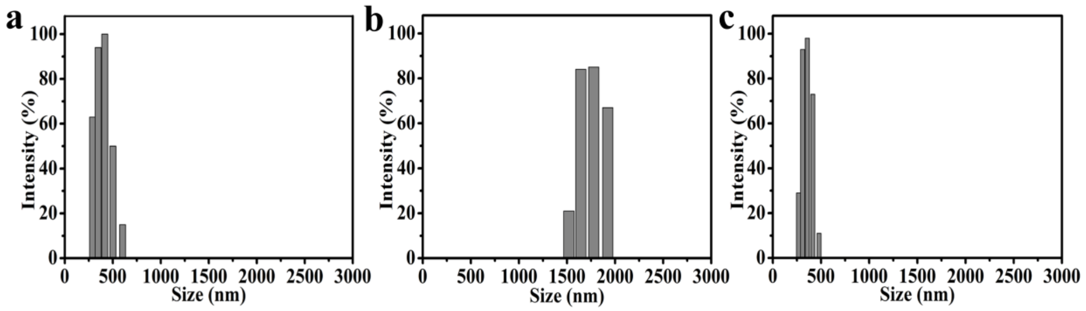

Figure S10. (**a**) Hydrodynamic size of C_3N_4 nanoribbons. (**b**) Hydrodynamic size of Cu^{2+}-C_3N_4 nanoribbon complex. (c) Hydrodynamic size of C_3N_4 nanoribbons after the $C_6H_5O_7^{3-}$ was added into Cu^{2+}-C_3N_4 nanoribbon solution.

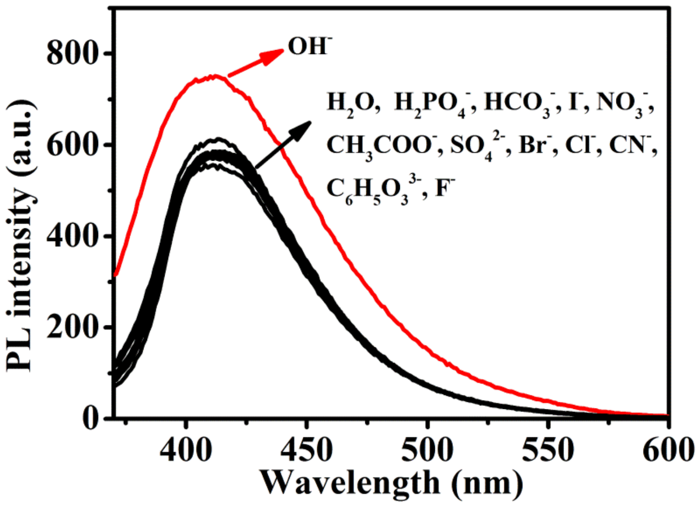

Figure S11. The fluorescence responses of C_3N_4 nanoribbons in aqueous solution upon addition of different anions (Br^-, $C_6H_5O_7^{3-}$, Cl^-, CN^-, F^-, $H_2PO_4^-$, HCO_3^-, I^-, NO_3^-, OH^-, CH_3COO^-, and SO_4^{2-}) (final concentration: 1 mM).

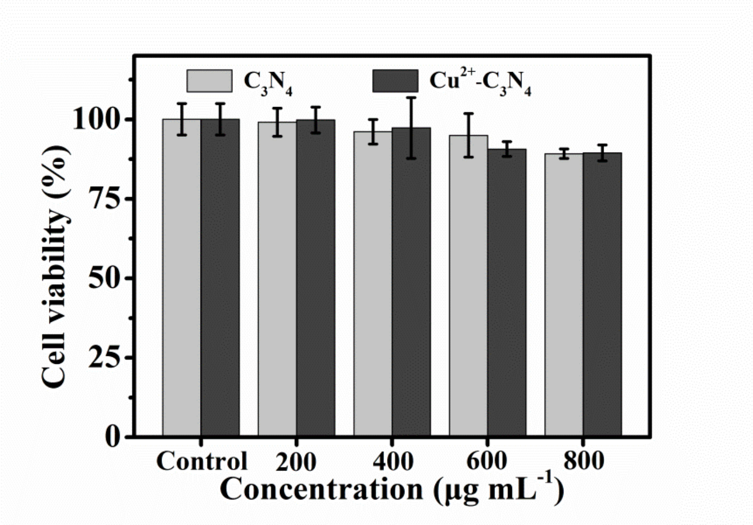

Figure S12. Cell viability of HeLa cells incubated with various concentrations of C_3N_4 nanoribbons (grey) or Cu^{2+}-C_3N_4 nanoribbon complex (black) for 24 h.

Table S1. Comparison of fluorescent citrate sensors.

Materials	Detection limit	Linear range	Response time	Reference
Coumarin	0.19 μM	0.1-0.5 μM	-	[6]
Rhodamine	25 nM	0.1-50 μM	1 min	[7]
Diketoprrrolopyrrole	0.18 μM	0–40 μM	-	[8]
CdTe quantum dots	60 nM	0.67–133 μM	5 min	[9]
Boronate derived	10 nM	0~950 nM	15 min	[10]
Carbon nitride	0.78 μM	1–400 μM	20 s	This work

References

1. Yang, J.; Wu, X.; Li, X.; Liu, Y.; Gao, M.; Liu, X.; Kong, L.; Yang, S. Synthesis and characterization of nitrogen-rich carbon nitride nanobelts by pyrolysis of melamine. *Appl. Phys. A-Mater.* **2011**, *105*, 161.
2. Wang, H.; Qi, C.; He, W.; Wang, M.; Jiang, W.; Yin, H.; Ai, S. A sensitive photoelectrochemical immunoassay of n_6-methyladenosine based on dual-signal amplification strategy: Ru doped in SiO_2 nanosphere and carboxylated g-C_3N_4. *Biosens. Bioelectron.* **2018**, *99*, 281–288.
3. Tian, J.; Liu, Q.; Asiri, A.M.; Sun, X.; He, Y. Ultrathin graphitic c3n4 nanofibers: Hydrolysis-driven top-down rapid synthesis and application as a novel fluorosensor for rapid, sensitive, and selective detection of Fe^{3+}. *Sens. Actuators B* **2015**, *216*, 453–460.
4. Xiang, Q.; Yu, J.; Jaroniec, M. Preparation and enhanced visible-light photocatalytic H_2-production activity of graphene/C_3N_4 composites. *J. Phys. Chem. C* **2011**, *115*, 7355–7363.
5. Tahir, M.; Cao, C.; Mahmood, N.; Butt, F.K.; Mahmood, A.; Idrees, F.; Hussain, S.; Tanveer, M.; Ali, Z.; Aslam, I. Multifunctional g-C_3N_4 nanofibers: A template-free fabrication and enhanced optical, electrochemical, and photocatalyst properties. *ACS Appl. Mater. Interfaces* **2014**, *6*, 1258–1265.
6. Liu, Z.; Devaraj, S.; Yang, C.; Yen, Y. A new selective chromogenic and fluorogenic sensor for citrate ion. *Sens. Actuators B* **2012**, *174*, 555–562.
7. Li, C.; Zhou, Y.; Li, Y.; Kong, X.; Zou, C.; Weng, C. Colorimetric and fluorescent chemosensor for citrate based on a rhodamine and Pb^{2+} complex in aqueous solution. *Anal. Chim. Acta.* **2013**, *774*, 79–84.
8. Hang, Y.; Wang, J.; Jiang, T.; Lu, N.; Hua, J. Diketopyrrolopyrrole-based ratiometric/turn-on fluorescent chemosensors for citrate detection in the near-infrared region by an aggregation-induced emission mechanism. *Anal. Chem.* **2016**, *88*, 1696–1703.
9. Zhuo, S.; Gong, J.; Zhang, P.; Zhu, C. High-throughput and rapid fluorescent visualization sensor of urinary citrate by CdTe quantum dots. *Talanta* **2015**, *141*, 21–25.
10. Rajalakshmi, K.; Nam, Y.; Selvaraj, M.; Lee, Y.; Lee, K. Metal free bioimaging reagent for intracellular citrate in prostate cancer cells using aryl boronate derivative. *Sens. Actuators B* **2018**, *259*, 90–96.

© 2018 by the authors. Submitted for possible open access publication under the terms and conditions of the Creative Commons Attribution (CC BY) license (http://creativecommons.org/licenses/by/4.0/).
